# Multi-trial analysis of HIV-1 envelope gp41-reactive antibodies among global recipients of candidate HIV-1 vaccines

**DOI:** 10.3389/fimmu.2022.983313

**Published:** 2022-10-12

**Authors:** Koshlan Mayer-Blackwell, Andrew M. Johnson, Nicole Potchen, Simon S. Minot, Jack Heptinstall, Kelly Seaton, Sheetal Sawant, Xiaoying Shen, Georgia D. Tomaras, Andrew Fiore-Gartland, James G. Kublin

**Affiliations:** ^1^ Vaccine and Infectious Disease Division, Fred Hutchinson Cancer Research Center, Seattle, WA, United States; ^2^ Center for Human Systems Immunology, Duke University, Durham, NC, United States; ^3^ Department of Surgery, Duke University, Durham, NC, United States

**Keywords:** human immunodeficiency virus (HIV), vaccines, antibody, microbiota, clinical trial, cross-reacting antibodies

## Abstract

Many participants in HIV-1 vaccine trials, who have not previously been exposed to or vaccinated against HIV-1, display serum immunoglobulin antibodies that bind the gp41 region of HIV-1 envelope prior to vaccination. Previous studies have hypothesized that these pre-existing antibodies may be cross-reactive and may skew future vaccine responses. In 12 large studies conducted by the HIV Vaccine Trial Network (HVTN) (n=1470 individuals), we find wide variation among participants in the pre-vaccine levels of gp41-reactive antibodies as measured by the binding antibody multiplex assay (BAMA). In the absence of exposure to the gp41 immunogen, anti-gp41 IgG levels were temporally stable over 26-52 weeks in repeated measures of placebo recipients. The analysis revealed that the geometric mean of pre-vaccine anti-gp41 IgG response was greater among participants in South Africa compared with participants in the United States. With gene-level metagenomic sequencing of pre-vaccination fecal samples collected from participants in one trial (HVTN 106), we detected positive associations between pre-vaccine anti-gp41 IgG and abundance of genes from multiple taxa in the *Eubacteriales* order. The genes most strongly associated with higher baseline anti-gp41 IgG mapped to a clade containing *Blautia wexlerae* and closely related strains. In trials with vaccine products containing the full or partial portion of gp41 immunogen alongside a gp120 immunogen, we did not find evidence that individuals with higher baseline anti-gp41 IgG had different levels of anti-gp120 IgG after vaccination compared to individuals with lower pre-vaccine anti-gp41 levels (pooled estimate of standardized mean difference -0.01 with a 95% CI [-0.37; 0.34]).

## Introduction

The trimeric HIV-1 envelope glycoprotein consists of two glycoprotein subunits: gp120 and gp41. The transmembrane gp41 plays multiple critical roles in viral infectivity. Its fusion domain binds the host T cell and an energetically favorable conformational change during its activation drives fusion of the viral and host membranes (1). The glycoprotein’s multiple conformational states permit multiple antibody targets (2). Early research to determine epitopes targeted by monoclonal antibodies isolated from HIV-1 patients identified multiple binding sites; immunodominant domains eliciting ‘cluster 1’ type antibodies target the C-C’ loop peptides near cysteines at HXB2 reference positions 598 and 604 (3) and are common following initial infection (4). Infection elicits a rapid increase in these cluster 1 type anti-gp41 IgG and IgM in humans with a median onset of 13 days following infection compared to 18 days for anti-p24 and 28 days for anti-gp120 antibodies. In a majority of subjects IgM anti-gp41 was detected at the same time as IgG and IgA, which is uncharacteristic of antibody responses to a novel infection. Initial anti-gp41 antibodies are typically non-neutralizing and were not associated with control of viral load in natural infection (4). However, more recently it was observed that the rate of increase in the combined anti-gp41 IgM and IgG antibody concentration was strongly correlated with the initial rate of HIV-1 infectivity decay, suggesting a role of anti-gp41 antibody early in natural infection (5). In the context of chronic HIV infection, neutralizing anti-gp41 antibodies may develop (6, 7) and gp41 may also be an effective target for antibody-dependent cellular cytotoxicity (8).

Vaccines with a gp41 component induce robust anti-gp41 responses. A gp41 immunogen has been included in several HIV-1 vaccine candidates either as a component of the DNA prime (e.g., HVTN 098) or as a component of a viral vector (e.g., HVTN 204 and HVTN 505); notably, the RV144 vaccine regimen, which showed partial efficacy, included only the transmembrane domain of gp41 in the ALVAC-prime and did not include gp41 in the gp120 protein boost. By contrast to RV144, the C terminal and transmembrane domain portions of gp41 were included in both the DNA and the recombinant adenovirus-vectored vaccine of the HVTN 505 regimen. In that study, gp120 and gp41-reactive IgG antibodies, in combination with Env-specific CD8+ T cell responses, were associated with reduced risk of infection; however, the presence of baseline anti-gp41 IgG was not associated with HIV-1 risk (9). A subset of monoclonal anti-gp41 antibodies isolated from HVTN 505 vaccine recipients were shown to cross-react with bacterial proteins in the intestinal microbiota of humans (10, 11) and rhesus macaques (12), leading to the hypothesis that an immunodominant response primed by pre-existing gp41 cross-reactive B cells may divert some of the immune system’s capacity from maturing gp120-specific B cells (10). Specifically, a possible cross-reactive epitope was proposed in the HR1 region of gp41 (HXB2 555-559), with the LLRAIE amino-acid sequence motif having similarity with amino-acid sequences in bacterial RNA polymerase and pyruvate flavodoxin oxidoreductase proteins, which are common in the intestinal microbiome (IM). Additional investigations identified levels of IgG antibodies to gp41 at baseline to be associated with clusters of family level microbial taxa in the HVTN 096 study (13). Moreover, Williams et al. showed that in HVTN 505 Env-reactive vaccine-induced antibodies were dominantly reactive to gp41 relative to gp120. Potential to divert development of gp120-directed antibodies is of high-concern because neutralization is commonly achieved through targeting of regions of Env outside of gp41, and the protection observed in the RV144 trial was most strongly associated with antibodies targeting the V1V2 region in the gp120 region of Env (14–17).

Given the inconclusive role of gp41 cross-reactive antibodies in modulating overall response to vaccination, we examined more HVTN trials for evidence of pre-vaccination anti-gp41 antibodies. In HVTN trials, antibody response is primarily measured by a validated and Good Clinical Laboratory Practice (GCLP) compliant multiplex binding antibody assay (BAMA); participants’ pre-vaccine values are used as a comparator to measure the magnitude of vaccine-elicited antibodies. Pre-vaccination and placebo assay values suggest that there is a high prevalence of gp41-reactive antibodies at baseline, across a diverse set of participants and geographies. Although there is considerable variation in detectable anti-gp41 IgG, we found that these responses are temporally stable within participants. We also found that pre-vaccine cross-reactive anti-gp41 antibody responses were greater among trial participants in South African versus United States; one possible explanation is that diet- or environment-driven differences in the maintenance of specific components of the intestinal microbiome could play a role. In a recent HVTN trial, volunteers provided fecal microbiome samples prior to vaccination, and we generated gene-level microbiome data that suggest that pre-vaccination anti-gp41 IgG positively associated with relative abundance of genes from Enterobacteria *(Blautia* sp. and *Blautia wexlerae*), consistent with a possible role for the human intestinal microbiome in modulating levels of antibodies that cross-react with HIV-1 gp41.

## Methods

### Study selection and binding antibody measurement

HIV Trials Network (HVTN) studies with more than 40 participants were considered for inclusion in the multi-trial analysis. The primary criterion was the availability of anti-gp41 IgG measurements measured near the common 50-fold dilution by Binding Antibody Multiplex Assay (BAMA). All participants were determined to be seronegative for HIV-1 and HIV-2 prior to enrollment using an approved enzyme immunoassay (EIA) or chemiluminescent microparticle immunoassay (CMIA) for antibody against HIV antigen. Participants in the phase 1 studies (HVTN 096, 098, 106, 122, 097, 107, 100, 111) were determined to be at low risk of HIV infection using criteria applied to the number and type of sexual behaviors reported in the previous 6 – 12 months.

Data includes measurement of background fluorescence and fluorescence on beads absent antigen. Blank bead with background fluorescent subtraction is recorded as [fi_bkgd_blank]. We excluded all participants with blank bead binding greater than 500 MFI that would overlap with low-level pre-vaccine antigen specific signals (**Table 1**). Antigen bead fluorescence with background fluorescent subtraction is recorded [fi_bkgd]. Thus, [fi_bkgd] - [fi_bkgd_blank] records fluorescence signals attributable to antigen specific antibody binding. The assay’s lower limit of detection is 100 MFI, thus we considered [fi_bkgd] - [fi_bkgd_blank] that exceeded double the machine limit (i.e., 200 MFI) as evidence of cross-reactive antibodies. Data meeting baseline gp41 inclusion criteria included patient samples from 11 countries; however, more than half of the data came from sites in the United States and South Africa. Number of trial participants per country are summarized in **Table S1**. Linear peptide binding measurements obtained from peptide microarray assay were normalized (using R package “pepStat”) median fluorescence intensity values of triplicate spots on array slides.

**Table 1:** Number of participant with pre- and post-vaccine gp41 BAMA measurements meeting QC requirements.

	Trial	Number of Participants with Dilution 50 anti-gp41 IgG at Pre-Vaccine Timepoint			Total Included	Excluded Because of QC Reason		Excluded Because anti-gp41 IgG > 500 MFI		Pre-Vaccine anti-gp41 (IQR)	
		Female	Male	Total		Female	Male	Female	Male	Median [IQR]	Log10 Median [IQR]
HVTN	96	44	44	88	80	1	1	1	5	270 [133,548]	2.4 [2.1,2.7]
HVTN	97	38	42	80	79	0	0	1	0	171 [61,427]	2.2 [1.8,2.6]
HVTN	98	38	46	84	54	5	4	11	10	181 [85,400]	2.3 [1.9,2.6]
HVTN	100	95	132	227	200	0	0	12	15	294 [142,627]	2.5 [2.2,2.8]
HVTN	106	38	55	93	77	1	4	5	6	219 [152,552]	2.3 [2.2,2.7]
HVTN	107	64	48	112	92	3	4	6	7	475 [302,991]	2.7 [2.5,3]
HVTN	111	69	67	136	97	8	10	8	13	315 [164,652]	2.5 [2.2,2.8]
HVTN	122	26	12	38	27	4	1	4	2	408 [192,626]	2.6 [2.3,2.8]
RV	144	97	138	235	219	0	0	10	6	159 [81,343]	2.2 [1.9,2.5]
HVTN	204	201	222	423	339	11	11	30	32	653 [333,1737]	2.8 [2.5,3.2]
HVTN	205	33	42	75	62	2	0	3	8	324 [102,623]	2.5 [2,2.8]
HVTN	505		189	189	144		19		26	195 [61,326]	2.3 [1.8,2.5]

Exclusions were due to QC Flags (N): High Blank (>5000 MFI), Sample and Baseline Visits (53), High Blank (>5000 MFI), Sample Visit (15), HIV Infection (13), High Blank (>5000 MFI), Baseline Visit (5). High Background (>5000), Sample Visit (1), High Background (>5000), Sample and Baseline Visits (1), High Antigen (>6500 MFI*), Baseline Visit (1).

### gp41 vaccine immunogens

We divided trials into two categories: (i) those with vaccines containing the full or partial gp41 as a component of the HIV-1 envelope (i.e., containing gp41, gp140, gp150, or gp160 immunogens) and (ii) those with vaccines containing only gp120 protein plus the 20 amino acid residues of the gp41 forming the transmembrane domain (HXB2 reference positions 685-704). Partial gp41 immunogens such as gp140 and gp150, but not the transmembrane domain, were expected to elicit a significant gp41 response. This was confirmed by observation of pre-versus post-gp41 responses.

The form of gp41 and delivery vector varied by trial: HVTN 96 tested a plasmid DNA-HIV-PT123 encoding a clade C ZM96 Env and NYVAC a highly attenuated vaccinia virus expressing HIV clade C ZM96 Env gp140. HVTN 98 tested PENNVAX-GP, a circular, double stranded, deoxyribonucleic acid consisting of expression plasmids that encode synthetic HIV-1 multiclade consensus Gag, Pol and Env proteins. HVTN 106 tested a DNA plasmid later primed with Modified Vaccinia Ankara (MVA-CMDR) Modified Vaccinia Ankara (MVA), a recombinant, live attenuated vaccinia virus vectored vaccine that has been genetically engineered to express HIV-1 gp150 (Subtype E, isolate CM235). HVTN 204 and HVTN 505 tested a multiclade DNA + Recombinant Adenovirus Type 5 (DNA/rAd5) vaccine containing trivalent mixture of clade A, B and C *env* gp140 genes contained both gp120 and gp41 ectodomain components (see **Table 2**).

**Table 2:** Vaccine product description by trial.

Trial	Vaccine Product	Env	Clade
Vaccines with the full or partial gp41 immunogen that elicited anti-gp41 response			
HVTN 96	Plasmid DNA-HIV-PT123 encoding a clade C ZM96 Env and NYVAC a highly attenuated vaccinia virus expressing HIV clade C ZM96 Env gp140.	gp140	C
HVTN 98	PENNVAX-GP, a circular, double stranded, deoxyribonucleic acid consisting of expression plasmids that encode synthetic HIV-1 multiclade consensus Gag, Pol and Env proteins. Env A/Env C.: PENNVAX^®^ -GP will require the admixing of Env A/Env C	gp160	A,C
HVTN 106	DNA plasmids containing gp160 followed by Modified Vaccinia Ankara (MVA-CMDR), a recombinant, live attenuated vaccinia virus vectored vaccine that has been genetically engineered to express HIV-1 gp150 (Subtype E, isolate CM235).	gp160 (DNA) + gp150 (MVA)	E
HVTN 122	3 doses of protein gp145 C.6980 100mcg or 300mcg with Alum	gp145	C
HVTN 204	Multiclade DNA + Recombinant Adenovirus Type 5 (DNA/rAd5) vaccine containing trivalent mixture of clade A, B and C env gp140 genes contained both gp120 and gp41 ectodomain components.	gp140	A,B,C
HVTN 205	DNA vaccine: pGA2/JS7 DNA is a 9.5 kb plasmid DNA expressing the HIV-1proteins Gag, PR, RT, Env, Tat, Rev, and Vpu, from a single transcript. MVA vaccine: Recombinant modified vaccinia Ankara/HIV clade B gag-pol-env (MVA/HIV62) is a highly attenuated vaccinia virus expressing HIV-1 gag, pol, and env genes from the same sequences used to construct the JS7 DNA.	gp160(DNA) + gp160(MVA)	B
HVTN 505	Multiclade DNA + Recombinant Adenovirus Type 5 (DNA/rAd5) vaccine containing trivalent mixture of clade A, B and C env gp140 genes contained both gp120 and gp41 ectodomain components.	gp140	A,B,C
**Vaccines lacking gp41 immunogen that did not elicit anit-gp41 response**			
HVTN 97	Combined ALVAC-HIV (vCP1521) expressing HIV-1 clade E env AIDSVAX B/E a bivalent HIV gp120 glycoprotein with subtype B (MN) and subtype E (A244).	gp120*	E, B/E
HVTN 107	ALVAC mo(0,1,3,6,12) + Bivalent Subtype C gp120/MF59 mo(3,6,12)	gp120*	C
HVTN 100	ALVAC mo(0,1) + ALVAC & gp120 & MF59 mo(3,6,12)	gp120*	E, B/E
HVTN 111	gp120/MF59 protein clade C	gp120	C
RV144	RV144 evaluated the heterologous prime-boost combination of canary pox prime ALVAC-HIV (vCP1521), expressing clade E env and clade B gag and pro, followed by the AIDSVAX clades B/E gp120 protein boost in a community population of HIV- 1 uninfected participants in Thailand	gp120*	E, B/E

*ALVAC contains the transmembrane domain of gp41 in addition to full length gp120.

### Statistical methods

#### Country level variation

Country level variations were compared by combining results across protocols and computing the geometric mean of results for individuals within each country. Robustness of differences between measured levels of pre-existing gp41 cross-reactive IgG prior to vaccination in the United States and South Africa, was assessed using ordinary least square regression with log10-transformed, background subtracted IgG fluorescent signal regressed against age, sex assigned at birth, protocol, and country of the study site as covariates.

The BAMA assay is validated for the specific purpose of direct comparisons across batches and over time; all antigen batches passed pre-specified Levy-Jennings (LJ) acceptance criteria (control samples within 2-fold) for the datasets that were included. The significant difference between South Africa and United States was robust to the removal of South African HVTN 107 trial data, which was generated with gp41 antigen lot J1-17M1, which was within the LJ criteria but tracked higher than other lots. Anti-gp41 IgG levels in HVTN 107, using the J1-17M1 antigen lots, were higher than in four other separate South African trials using a separate antigen lot, so we excluded it from country-by-country comparisons. Median participant age is lower in South African trial sites compared to U.S. trial sites, so to ensure that the difference we observed could not simply be explained by compositional effects, we subset the data to include all participants in U.S. and South African sites (including and excluding HVTN 107) and regressed pre-vaccine anti-gp41 IgG (log10 transformed) against age, sex, country, and trial protocol (**Table S2**).

### Group difference vaccine-matched responses

For group comparisons, we formed groups in each protocol by splitting about the median baseline anti-gp41 value. Where multiple post-vaccine sample visits exist, we selected visit samples with the highest median gp120 signal. We standardized all post-vaccine responses by subtracting the within protocol mean and dividing by standard deviation. Where a study measured multiple vaccine-matched gp120 antigens, we took the mean of standardized antigen response. Group comparisons are assessed *via* an unpaired two-sided t-test. Because of between-study heterogeneity in vaccine regimen and study population, we used a random-effects model to estimate a pooled effect size. We used a restricted maximum likelihood estimator (18) to calculate the between-study heterogeneity variance. To estimate confidence intervals around the pooled effect mean standard difference, we used the Knapp-Hartung adjustments (19). Pooled estimates and forest plots generated with the *meta* (20) and *dmetar* (21) packages in the R 4.1.2 statistical environment.

### Metagenomic samples and sequencing

Fecal samples from a subset of HVTN 106 participants (n = 37) were collected at trial sites on a voluntary basis. Samples were frozen and stored at -20C. Samples were thawed, and DNA extracted with mechanical lysis to extract total DNA. Libraries were prepared using the QIAGEN microbial DNA extraction kit according to manufacturer, and libraries were paired-end 2x150bp sequenced using a HI-Seq Illumina instrument. The number of reads per specimen ranged from 4.2 to 27.3 million (median 14.7 million).

#### Metagenomic analysis

Reads were analyzed using the geneshot pipeline (v0.8.1) which is described in detail in (22). Briefly, geneshot addresses the problem of high dimensionality in gene-level metagenome analysis, by assembling reads from each specimen into contigs *via de novo* assembly using *megahit* (23); identifying protein-coding sequences within those contigs *with prodigal* (24); deduplicating similar coding sequences across all specimens using *mmseqs2* (25); aligning reads to that gene collection with *diamond* (26), and subsequently grouping genes based on their correlated levels of relative abundance across specimens, which is described in more detail in (27). These groups, referred to as co-abundant genes groups (CAGs), vary in size and may comprise conserved core portions of the genome defining a bacterial or archeal species or represent mobile genetic elements which may contribute to strain level genetic diversity. CAGs are not constrained by taxonomic annotations, but taxonomic origin of genes within each CAG are inferred based on alignment to RefSeq genomes. A CAG is assigned a tentative taxonomic association based on the most common taxa assigned to its component genes.

We specified a formula for statistical analysis, where the number of reads mapping to each CAG was modeled using a beta-binomial model with the formula (sex + age + log10-transformed anti-gp41 IgG) describing the logit of the expected relative abundance of the CAG, using maximum likelihood parameter estimation implemented in the software corncob (28). Furthermore, taxon abundance values are calculated by aggregating over all CAGs for which any constituent gene is taxonomically annotated. In practice, the benefit of this approach is to reduce the number of total hypotheses considered by only considering associations with taxa and genes found within an appreciable number of specimens in the study.

## Results

### Prevalence of anti-gp41 immunoglobulin in a global multi-trial analysis of randomized, placebo-controlled trials

**Figure 1:**
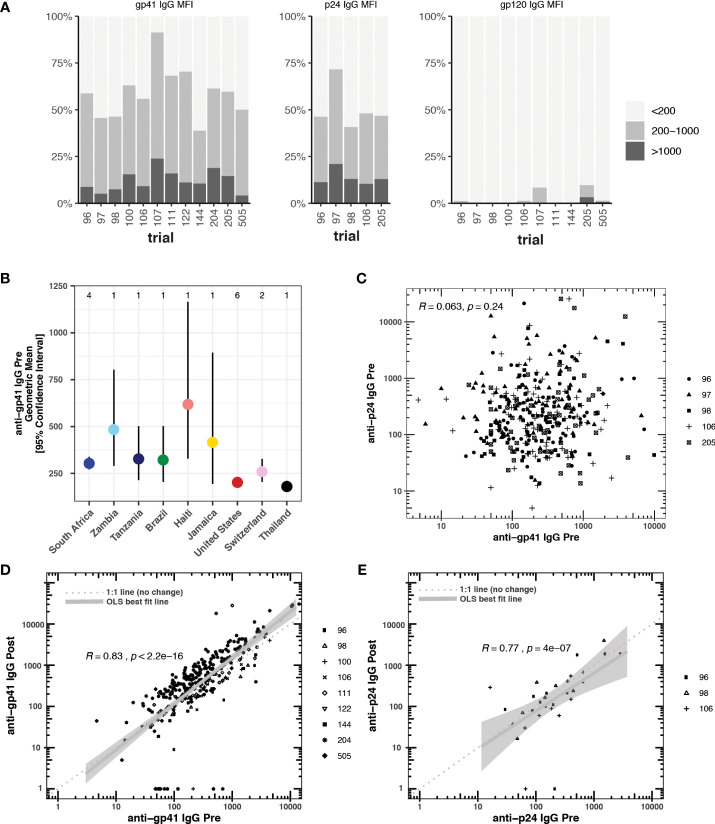
**(A)** Blank bead subtracted pre-vaccine cross-reactive anti-gp41, anti-p24, and anti-gp120 IgG responses measured by BAMA (mean fluorescence intensity, MFI) prior to vaccination in 12 HTVN trials. Responders with blank bead signals greater than 500 MFI were excluded from the analysis. **(B)** Country-level geometric means and 95% confidence intervals for pre-vaccine anti-gp41 IgG. The number of studies considered in each country is shown at the top of the plot. **(C)** Pre-vaccine anti-gp41 IgG vs pre-vaccine anti-p24 IgG, **(D)** Pre-vaccine anti-gp41 IgG vs. post-vaccine anti-gp41 IgG in placebo recipients (n= 314). **(E)** Pre-vaccine anti-p24 IgG vs. post-vaccine anti-p24 IgG in placebo recipients (n = 31).

We selected studies with more than 40 participants and pre- and post-vaccine anti-gp41 IgG measurements near the most common 50-fold dilution level (**Table 1**). We sought to include studies with participants from multiple study sites to avoid confounding geographical variation with possible batch effects in the sample collection and processing; however, several studies were conducted entirely within a single country (**Table S2**). In 12 HVTN vaccine trials, we found that anti-gp41 IgG antibodies in serum were commonly found prior to vaccination in a diverse set of trial participants across age, sex, and geography. After filtering-out samples with a high level of IgG binding to the negative control “blank” bead (i.e., bead without antigen), we examined pre-vaccine anti-gp41 IgG responses in 1462 trial participants. Among these participants, anti-gp41 IgG antibodies were detectable (MFI > 200) in 58% of participants. Additionally, 13% of participants had more appreciable pre-vaccine anti-gp41 IgG (MFI > 1000). By contrast, detectable anti-gp120 IgG prior to vaccination was rare, detectable in 1.5% (MFI > 200) of participants (**Figure 1A**). In general, the geometric mean of gp41 responses was higher in pre-vaccine samples of South African study participants (303, [271, 338] 95% CI; n =502, 4 studies) than participants in Thailand (179 [152, 211] 95% CI, n =219, 1 study) or the United States (203 [181, 226] 95% CI; n = 475, 6 studies) (**Figure 1B**), however, variability between individuals was high regardless of geographic region (**Figure S1**). The differences in geometric means were statistically significant, with 1.49-fold higher (95% CI 1.28 to 1.75) levels in South Africa versus the United States and 1.69-fold higher versus Thailand (95% CI, 1.39 to 2.06).

We found a significant negative association for participants located in the United States relative to South Africa when we regressed pre-vaccine anti-gp41 IgG (log10-transformed) against age, sex, country, and trial protocol (**Table S2**). Only one trial, HVTN 204, included participants at both trial sites within the United States and South Africa. When we examined this trial in isolation, controlling for sex and age, we also found a significantly lower level of pre-vaccine anti-gp41 IgG among U.S. participants (**Table S3**).

**Figure 2:**
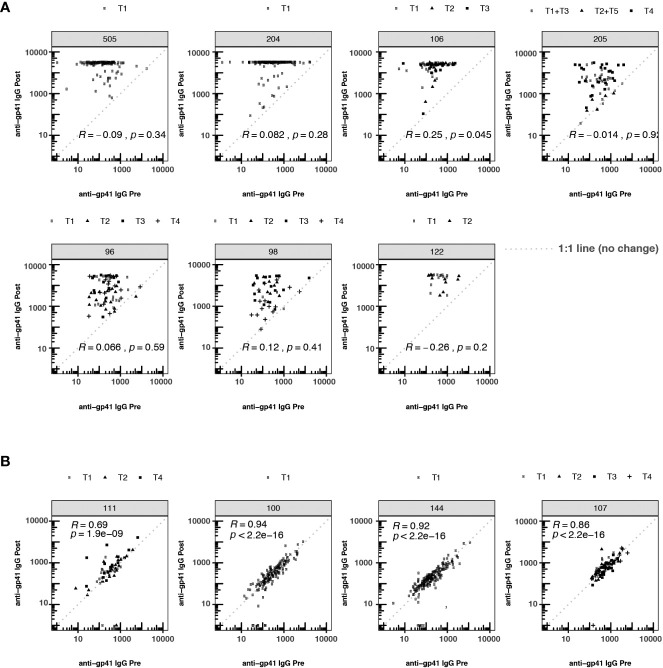
Participant-matched anti-gp41 IgG (MFI) pre- vs. post-vaccine for participants receiving either **(A)** vaccines containing gp41 immunogens or **(B)** vaccines lacking gp41 immunogens. Consistency in response magnitude in panel **(B)**, among participants receiving vaccines without gp41, shows the temporal stability of participants’ anti-gp41 cross-reactive response between measurements collected 26-52 weeks apart. The dashed 1:1 line represents no change between pre- and post-timepoints. T1-T5 indicates specific variation in vaccine dose regimen within a particular trial. Within each trial the nature of the gp41 immunogen was consistent across regimens.

Pre-vaccination antibodies against p24, an antigen on the HIV-1 structural capsid, were also common across five trials with available data (**Figure 1A**). The levels of anti-gp41 and anti-p24 antibodies were uncorrelated in those samples where both responses were measured (**Figure 1C**), suggesting that levels of anti-gp41 IgG reflect distinct antigenic recognition as opposed to some level of non-specific binding that may be reflective of the total immunoglobulin levels. Inspection of anti-gp41 responses in placebo recipients revealed that the magnitudes of anti-gp41 response were consistent between pre-vaccine and later trial visits (Rank correlation, ρ = 0.84), and follow-up measurements collected 26-52 weeks after the pre-vaccine visit (**Figure 1D**). Similar temporal stability in participants’ gp41 response was observed in vaccine trials where the study product lacked any of the immunodominant portions of the gp41 (HVTN111, HVTN100, RV144, HVTN107, **Figure 2**). Stability was also observed in anti-p24 IgG levels measured in placebo recipients (**Figure 1E**).

### Association of pre- and post-vaccination levels of anti-gp41 IgG

To investigate whether pre-existing gp41 antibodies predisposed participants to more immunodominant responses to gp41 after vaccination, we tested whether the magnitude of pre-vaccine responses was associated with the magnitude of post-vaccine responses. The post-vaccine anti-gp41 responses were robust in the HVTN 204, 505 and 122 trials, with most participants registering at the upper quantitative range of the fluorescence-based assay at the 1:50 dilution. Thus, discerning associations between pre- and post-vaccine responses was hindered for these trials by the limited range of observable post-vaccine variation (**Figure 2**). Nevertheless, in trials where gp41 measurements did vary within the quantitative range of the assay, we detected a positive association between pre- and post-vaccine anti-gp41, including in HVTN 106 (Rank correlation,ρ = 0.25, p-value = 0.045). Small positive, non-statistically significant rank-correlations were observed in HVTN 096 (ρ = 0.066), HVTN 204 (ρ = 0.082), and HVTN 098 (ρ = 0.12) (**Figure 2**).

### Lack of association between pre-vaccination anti-gp41 IgG and post-vaccination anti-gp120 IgG

**Figure 3:**
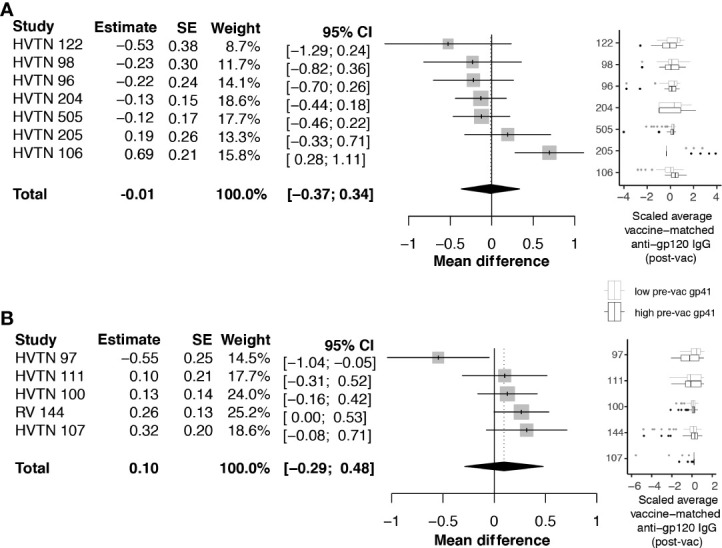
Difference in post-vaccine anti-gp120 IgG comparing trial participants with pre-vaccine anti-gp41 IgG level above vs. below the median level. Estimates and total pooled mean difference were estimated using random effects meta-analysis (see *Methods* for details). Models included either **(A)** protocols where vaccines included a gp41 immunogen, or **(B)** protocols where gp41 was not part of the study vaccine. Forest plots indicates estimated difference for each study, using size of the gray square to indicate study size, and horizontal line to indicate 95% CI. Boxplots to the left show distributions of scaled post-vaccine anti-gp120 averaged across vaccine matched antigens, where the box (IQR) and whiskers (+/- 1.5 IQR) show distribution of response among participants split above (black) and below (gray) the median pre-vaccine anti-gp41 response measured in each trial.

Next, we investigated whether pre-vaccination anti-gp41 IgG was associated with vaccine-matched gp120-specific responses. A negative association would be consistent with the hypothesis that gp41 immunodominance could act as a diversion from other envelope responses. For HVTN 505, HVTN122, HVTN 98, and HVTN 96, rank correlations between pre-vaccine anti-gp41 IgG and vaccine-matched gp120 antigens were negative, however the rank correlations were not statistically different from zero. We subsequently considered an analysis that split participants into two groups based on the per trial median pre-vaccine anti-gp41 IgG response. In trials with vaccine products containing the full or partial portion of gp41 we did not find evidence that the anti-gp120 vaccine responses were different among the high vs. low levels of anti-gp41 IgG at baseline; the pooled estimate of standardized mean difference was -0.01 with a 95% CI [-0.37; 0.34] (**Figure 3A**). HVTN 106 was an exception from the other trials in this regard. That is, individuals with high baseline anti-gp41 IgG mounted statistically significant greater mean anti-gp120 IgG responses following vaccination (standardized mean difference 0.69 [0.28; 1.11]). Excluding HVTN 106, the pooled estimate of the standardized mean difference is -0.13 [-0.31; 0.04] (**Figure S2**).

Considering the trials of vaccines that did not contain a gp41 immunogen beyond the 20 amino acid residues of the envelope transmembrane domain (HXB2 685-704), the standardized mean difference in anti-gp120 vaccine response between participants with high vs. low baseline anti-gp41 IgG was 0.10 with 95% CI [-.29; 0.48] (**Figure 3B**). These results do not provide evidence for the hypothesis that levels of pre-existing anti-gp41 antibody modify vaccine-induced anti-gp120 responses; however, there was considerable heterogeneity in the gp120 responses among individuals with high pre-vaccine gp41 IgG responses, including some non-response to gp120, leaving open the possibility that immune diversion might occur in a subset of participants. To further investigate, we regressed levels of anti-gp120 post-vaccine against pre-vaccine gp41 controlling for age and sex of each participant, but we did not find statistically significant associations (**Table S4**).

### Linear epitope mapping of pre-vaccine anti-gp41 IgG

The epitopes in gp41 that are targeted by pre-existing antibodies are unknown. Moreover, whether vaccinations tend to boost pre-existing anti-gp41 responses or elicit new responses is also an important question. Thus, we examined linear peptide antibody epitope mapping data from HVTN 106. The trial evaluated three vaccine regimens that each included three primes with DNA-encoded gp160 followed by a boost with a recombinant, live-attenuated modified vaccinia Ankara (MVA) virus expressing HIV-1 gp150 (isolate CM235). Linear epitope mapping was conducted prior to vaccination and 2 weeks after the MVA boost, providing an opportunity to observe whether anti-gp41 antibodies target the same linear epitopes before and after vaccination.

**Figure 4:**
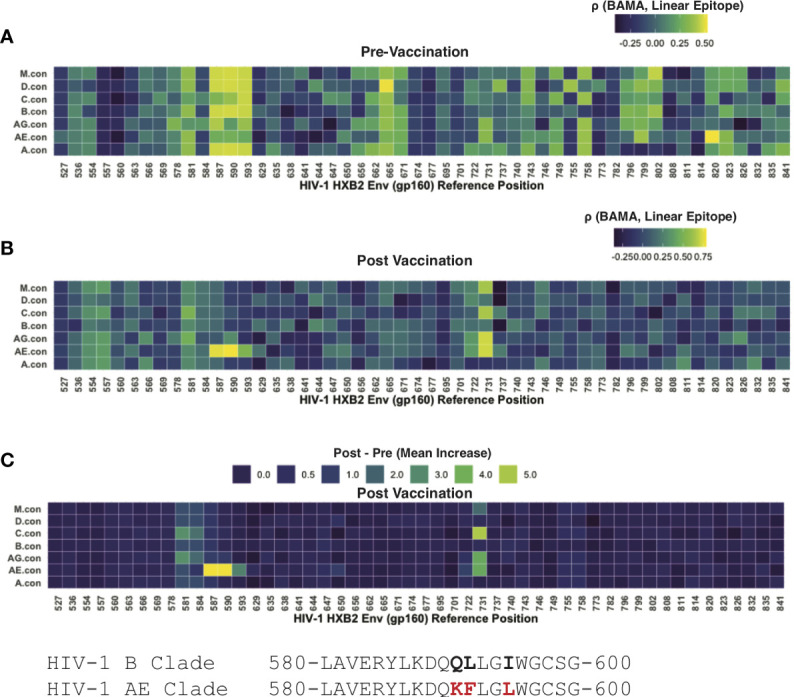
Linear epitope specific anti-gp41 Ig responses measured by linear epitope microarray from participants in HVTN 106. **(A)** Rank correlation between BAMA measured anti-gp41 IgG and linear epitope binding signal prior-to-vaccination. **(B)** Rank correlation between BAMA measured anti-gp41 IgG and linear epitope binding signal after-to-vaccination. **(C)** Mean increase in linear-epitope response signal.

Linear peptide arrays were designed using 15-mer peptides tiling the majority of the Env protein (with 12 AA overlap) (17, 29); peptides were derived seven consensus sequences representing each of the following: group M or subtypes D, C, B, AG, AE, A. Post-vaccination samples had high levels of antibody binding that was readily separable from background fluorescence, as measured from control spots containing no antigen. However, the pre-vaccination levels of most samples were near background levels. To interrogate these potential low-level responses, we leveraged paired data from participants whose samples were evaluated using both linear epitope mapping and whole antigen gp41 *via* BAMA. We hypothesized that correlation between the gp41 BAMA signal and binding to an individual linear peptide would be evidence of antibodies targeting that peptide, even in the absence of a strong absolute fluorescence signal. Using pre-vaccine samples, we found the strongest correlation of gp41 BAMA with linear epitopes spanning positions HVXB2 580-600 (**Figure 4A**). Notably, the correlation was strongest for variant peptides matching group M and subtypes D, B and A, and was weaker for the subtype AE variant peptides, which differed at three residues; the subtype AE peptides share these three substitutions with the subtype E vaccine immunogen (CM235 isolate; **Figure 4**)

After vaccination the correlation between linear epitope mapping and anti-gp41 IgG suggested a focusing in the gp41 target of antibodies. After vaccination there was no rank correlation observed between whole-antigen anti-gp41 IgG and linear epitopes matching the subtype M, D, B, and A peptides as had been observed prior to vaccination (**Figure 4B**); however, a strong positive correlation was observed for subtype AE consensus peptides spanning HXB2 580-597. Additionally, there was post-vaccine correlation with peptides spanning HXB2 575-588 and HXB2 725-738 (**Figure 4B**). Together these results shed light on the epitope specificities of anti-gp41 IgG both before and after vaccination and suggest that although the targeted region is similar after vaccination, the subtype variant recognition following vaccination is narrower.

We then examined the “LLRAI” region of gp41 at positions HXB2 555-560, which was previously proposed as a potential site of cross-reactivity (10). A strong signal of pre-vaccine antibodies recognizing this linear epitope was found in 6 of 35 participants. However, there was no evidence that anti-gp41 IgG antibodies specific to LLRAI were increased by vaccination, despite the sequence being conserved in the MVA vaccine immunogen (i.e., 01.CM235.MVA contains NLLRAIEAQQH). Also, the absence of study-wide correlation between whole-antigen gp41 (BAMA) and this linear epitope post-vaccination suggests that this was not a dominant vaccine-induced epitope response (**Figure 4C**). It may also be possible that anitbodies recognize a discontinuous or tertiary conformational epitope that would not be detected by peptide epitope mapping.

### Associations between pre-vaccine anti-gp41 IgG and the gut microbiome

**Figure 5:**
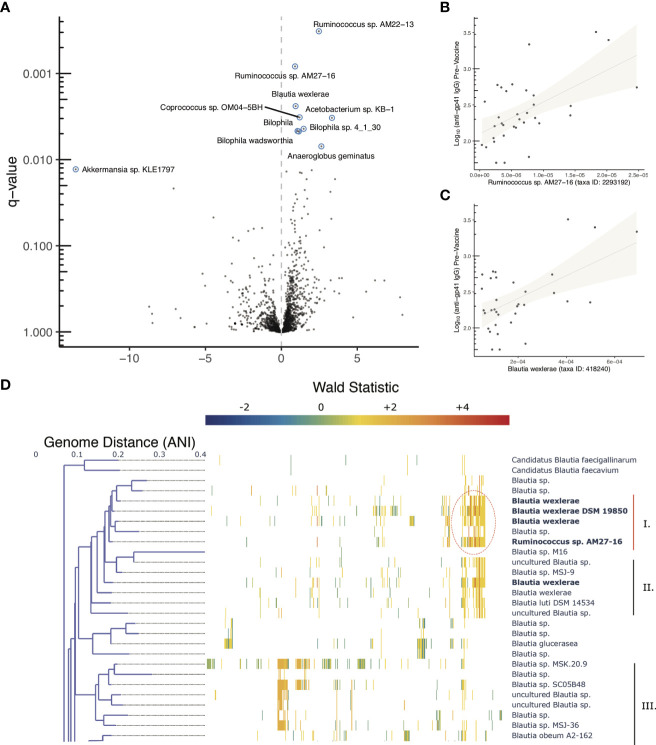
Microbiome associations with pre-vaccine gp41 IgG and Clustered co-abundant genes (CAGs) from fecal samples collected in HVTN 106. **(A)** Volcano plot of taxonomic features (the sum of all genes identified as part of any Clustered co-abundant genes (CAGs) assigned to a taxa, **(B, C)** Relative abundance of two of the most statistically significant taxa (x-axis) compared with pre-vaccine anti-gp41IgG (y-axis). **(D)** Fine scale mapping of genes identified in CAGs to genomes in the Blautia genus. Gene’s are mapped to genomes if they share 90% alignment identity at the amino acid level. The genes belonging to CAGs most consistently associated with anti-gp41 IgG pre vaccine have higher positive Wald statistics. **Figure S3** shows the full tree, containing members of the genus lacking genes associated with anti-gp41 IgG.

Using pre-vaccine fecal samples from HVTN 106 participants we examined whether features of the microbiome were associated with pre-vaccine magnitude of anti-gp41 IgG. Genomic DNA was extracted from fecal samples and deep sequenced for metagenomic analysis. Estimates of relative abundance of organisms were quantified by aligning short reads to the *de novo* assembled catalog of protein-coding genes, leveraging reference genomes deposited in NCBI (22); see Methods for details). A beta-binomial regression model was used to examine whether any taxonomic groups were associated with participant anti-gp41 IgG levels (BAMA) prior to vaccination, controlling for the age and sex-at-birth of each study participant (28). We found that the five most positively associated taxa and 14 of 27 significantly associated taxa (FDR < 0.05) were members of the order *Eubacteriales* (**Table S5**). Genes with primary annotations to the taxa *Ruminococcus AM27-16* (11 genes), *Blautia wexlerae* (330 genes), *Coprococcus* sp. *OM04-5BH* (17 genes), and *Lachnospira pectinoschiza* (27 genes) were the most strongly associated with pre-vaccine anti-gp41 (**Figures 5A–C**, **Table S5**). Upon close inspection the reference genome of the uncultivated *Ruminococcus AM27-16* manifests high genome-wide average nucleotide identity to multiple *Blautia wexlerae* genomes; this supports a taxonomic reassignment, which is consistent with many other strains historically mis-assigned as *Ruminococcus* that have subsequently been re-classified as members of the *Blautia* genus based on improved phylogenetic evidence (30). Large contiguous regions from the *Ruminococcus AM22-13* genome were not available for full comparison or conclusive taxonomic assignment, but aligned portions displayed 91.3% average nucleotide identity (ANI) with *Blautia wexlerae*, the closest matching type strain.

Genus level evaluations of *Blautia* have yielded conflicting results about the immunomodulatory role of the taxa (31). While the obligate anaerobic acetate-producing genus has been associated with preventing inflammation *via* promotion of regulatory T cells in some studies (32), in other studies abundance of the *Blautia* genus has been positively associated with irritable bowel syndrome and colitis (33, 34). Thus, to better understand granular strain-level associations with pre-existing anti-gp41 IgG, we aligned the genes of interest from HVN 106 fecal samples to 473 available *Blautia* complete and draft genomes to identify the strains that contained them. The association of each gene with anti-gp41 was quantified using a Wald metric, which captures both the direction and strength of association. There were two groups of genomes within the *Blautia genus* that contained genes associated with pre-vaccine anti-gp41 levels, with one clade containing the genes with the highest Wald scores (**Figure 5D**, **Figure S3**, genes with Wald scores > 4 appear in dark red). This clade comprises genomes that are close relatives to *Blautia wexlerae* and *Blautia luti*. The genes found to be most positively associated with anti-gp41 IgG in these taxa were absent in other Blautia species, suggesting a unique role of this clade relative to the genus at large. The subclade that included the isolate *Blautia wexlerae DSM 19850 (Clade I)* contained more of the highly associated anti-gp41 genes (Wald > 4), than the members of subclade containing *Blautia luti DSM 14534 (Clade II)* (**Figure 5D** *).* When we examined all available genomes with 90% average nucleodtide identity (ANI) to *Blautia wexlerae DSM 19850* we found that the most consistently anti-gp41 associated genes were detected in most, but not all of these close relative genomes (**Figure S3**). Therefore, future mechanistic testing of a hypothesized effect on anti-gp41 IgG could use an isolate such as *Blautia wexlerae DSM 19850*, belonging to that clade and containing the genes driving the observed statistical association.

We also observed positive associations between pre-vaccine anti-gp41 IgG and genes in CAGs annotated as *Deltaproteobacteria Bilophila* sp. *4_1_30* (25 genes), *Bilophila* (411 genes), and *Bilophila wadsworthia* (25 genes), however most genes in *Bilophila* reference genomes were found in gene clusters that were neutrally or negatively associated with anti-gp41 IgG, suggesting that genes found positively associated may be mobile genetic elements that are not be specific to this genus.

## Discussion

With this multi-trial analysis of 12 studies from 9 countries, we used data from a validated and GCLP compliant antibody binding assay to establish broad evidence of anti-gp41 IgG responses in individuals without known exposure to HIV-1 virus or vaccines. We also found anti-p24 IgG responses of similar prevalence and magnitude, though noted no such pre-existing responses to the gp120 portion of HIV-1 envelope. Previous studies of HIV-exposed, uninfected infants suggest that anti-gp41 IgG may develop as early as 18 months after birth, after marked decline in anti-gp41 maternal antibodies over the first 12 months (35). Across studies and individuals there was substantial heterogeneity in the levels of anti-gp41 and anti-p24 antibodies, though for both gp41 and p24, the quantitative levels of antibody were maintained over many months, lending further support that antibody binding may be antigen-specific, and not the result of technical variability or transient changes in non-specific binding. Though there was little evidence of systematic differences in anti-gp41 IgG across age and gender categories, we noted that there were significant differences across studies; while it remains possible that this is attributable to technical variability, we note that the assay and reagents met pre-specified validation criteria in all of the studies we included. When we attempted to account for trial-specific effects in our sensitivity analyses, there remained country-level effects – across studies – that were statistically significant. A limitation of the analysis is that there are many unobserved factors that could contribute to or be associated with differences in pre-existing anti-gp41 IgG levels. However, the results suggest that there may be some element of population genetics, diet, environmental exposures, or other geographic factors that are associated with the magnitude of pre-existing anti-gp41 responses. Given the previous evidence that anti-gp41 IgG cross-recognizes antigens from the intestinal microbiome, we note that the geographical heterogeneity could be directly related to dietary or environmental exposures that influence the human microbiome and differentiate geographic regions. Since all participants were determined to be HIV-1 seronegative at enrollment, and since all participants in the phase 1 trials (HVTN 096, 098, 106, 122, 097, 107, 100, 111) were determined to be at low risk of HIV infection, it is unlikely that prior, unknown exposure to the virus could explain the higher levels of pre-vaccine anti-gp41 antibody in South Africa.

To pursue the hypothesis that features of the intestinal microbiome modulate pre-vaccine levels of anti-gp41 IgG, we sequenced fecal microbiome samples from a single study with participants having a wide range of pre-existing gp41 IgG from the United States and Switzerland (HVTN 106). A limitation of our study is that fecal samples were not collected from the trial participants in South Africa where levels of pre-existing anti-gp41 IgG were highest. Though evidence from a single study is generally not sufficient to establish a mechanistic link between the microbiome and host traits, we reported statistically significant associations between several taxonomic features of participants’ intestinal microbiota and their levels of pre-existing anti-gp41 IgG. The most strongly associated features included positive associations with genes mapping to *Blautia wexlerie*. A linear peptide array analysis revealed that antibodies recognizing a few specific regions of gp41 were most strongly correlated with the whole-antigen gp41 readout prior to vaccination (**Figure 4A**); these could be considered as candidate regions of cross-reactivity. Together the findings support the need for further research into the possible antigen-specific cross-reactive mechanisms that may underlie the high prevalence and heterogeneity of anti-gp41 antibody responses.

Previous studies proposed a diversionary role of gp41 in vaccination against HIV-1 and identified that anti-gp41 responses were greater in magnitude than anti-gp120 responses after vaccination and that memory B-cells had a higher proportion of gp41-reactive clones (10, 12). However, these studies did not explicitly show that pre-vaccine anti-gp41 binding or induction of anti-gp41 responses is associated with lower anti-gp120 antibody responses following vaccination. In this cross-protocol analysis we did not find evidence that vaccine-matched anti-gp120 IgG responses were more robust among participants with lower pre-existing anti-gp41 IgG. There were weak associations that varied across studies and in most trials the pre-vaccine anti-gp41 response was uncorrelated with responses to other envelope antigens in the vaccine. Our findings across multiple trials suggest that pre-existing antibodies to one portion of the HIV envelope vaccine immunogen had little discernable impact on the development of antibodies to other epitopes on the same immunogen. However, it’s possible that the impact of pre-existing anti-gp41 IgG on the response to vaccination is context-dependent and it remains important to continue monitoring the role of pre-existing responses in future vaccine trials.

## Data availability statement

Data presented in this study are deposited in a FigShare repository 10.6084/m9.figshare.21178468. The raw microbial metagenomic reads supporting the conclusions of this article were deposited into the NCBI SRA under BioProject accession PRJNA886719; SRA accessions SRR21789197-233.

## Ethics statement

The studies involving human participants were reviewed and approved by local institutional review boards participating in each of the NIH NIAID HIV Vaccine Trials Network studies. The patients/participants provided their written informed consent to participate in this study.

## Author contributions

AF-G, KMB, JK, GT, XS, NP, SM and AJ conceived of the study and planned the analysis. JH, KS, SS, XS, and GT generated data. AF-G, KMB, SM and SS performed the analysis. AF-G, KMB, AJ, JK, GT and XS drafted the manuscript. All authors contributed meaningfully towards editing and finalizing the manuscript.

## Funding

This work was supported by the National Institutes of Health NIAID funded HIV Vaccine Trials Network (HVTN/ CoVPN) UM1 AI068618 (Lab); UM1 AI068635 (SDMC); UM1 AI068614 (LOC); and the Duke Center for AIDS Research (P30 AI064518).

## Acknowledgments

We thank Judith Lucas, Tara McNair, Michael Archibald, Tam Huynh, Kristy Long, Marcella Sarzotti-Kelsoe, Duke University. We give thanks to the HVTN Protocol Co-Chairs, Protocol Teams, Study Site Principal Investigators and staff and all the study volunteers who participated in these trials and whose commitments enabled this research.

## Conflict of interest

The authors declare that the research was conducted in the absence of any commercial or financial relationships that could be construed as a potential conflict of interest.

## Publisher’s note

All claims expressed in this article are solely those of the authors and do not necessarily represent those of their affiliated organizations, or those of the publisher, the editors and the reviewers. Any product that may be evaluated in this article, or claim that may be made by its manufacturer, is not guaranteed or endorsed by the publisher.
