# Supplementary material for: Multi-trial analysis of HIV-1 envelope gp41-reactive antibodies among global recipients of candidate HIV-1 vaccines
Source: Front Immunol. 2022 Oct 12;13:983313. doi: 10.3389/fimmu.2022.983313 (PMC9597301; doi:10.3389/fimmu.2022.983313)
Supplement: Supplementary file 1 [file DataSheet_1.pdf]

Multi-trial analysis of HIV-1 envelope gp41-reactive immunoglobulin antibodies among global recipients of candidate HIV-1 vaccines

Koshlan Mayer-Blackwell[1], Andrew M. Johnson[1], Nicole Potchen[1], Samuel S. Minot[1], Jack Heptinstall[2,3], Kelly Seaton[2,3], Sheetal Sawant S[2,3], Xiaoying Shen[2,3], Georgia D. Tomaras[2,3], Andrew-Fiore Gartland[1**], James Kublin[1**]

[1] Vaccine and Infectious Disease Division, Fred Hutchinson Cancer Research Center, Seattle, WA, USA

[2] Center for Human Systems Immunology, Duke University, Durham, NC, USA

[3] Department of Surgery, Duke University, Durham, NC, USA

** Corresponding Authors

Supplemental Figures

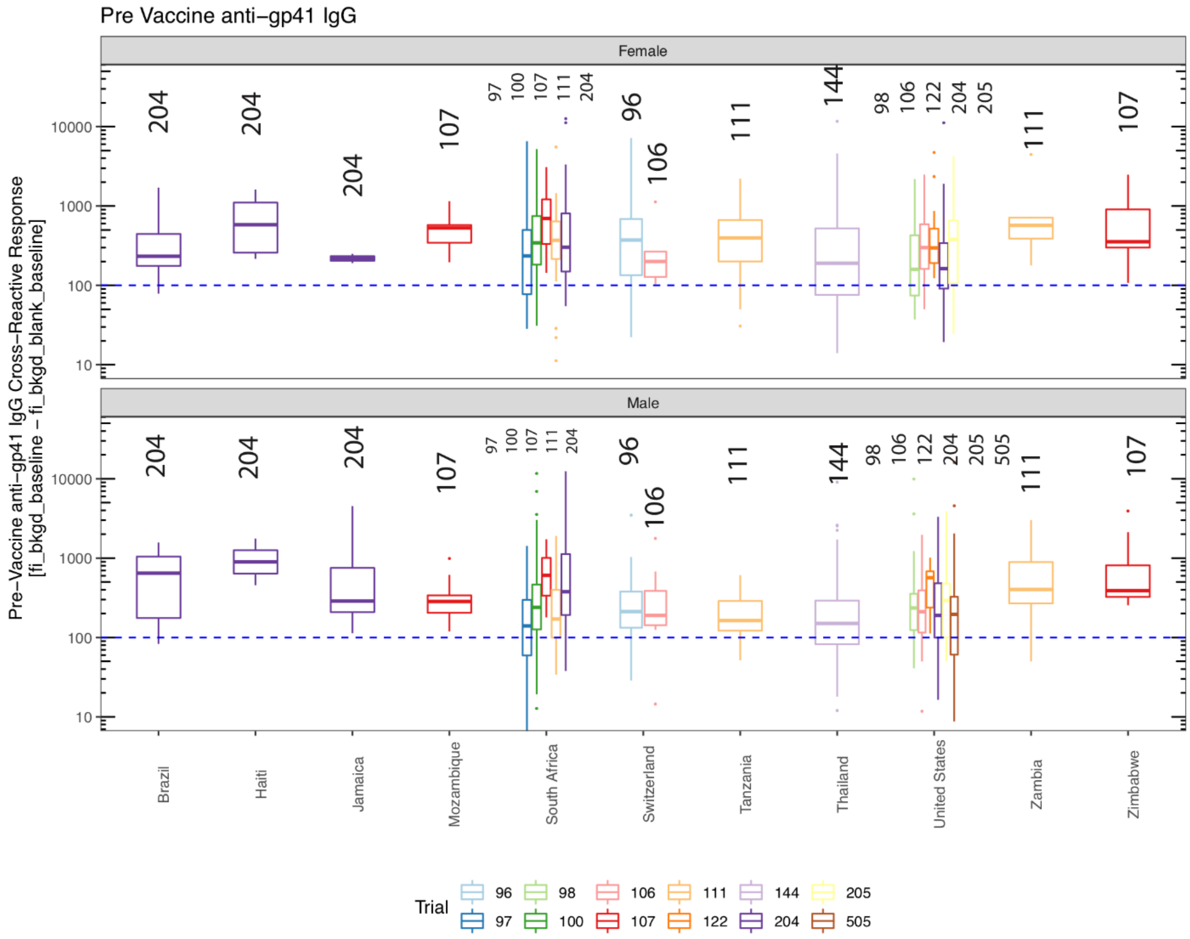

Figure S1. Heterogeneity in pre-vaccine cross-reactive anti-gp41 IgG response measured by BAMA by sex, country, and HVTN Trial number. Boxplots indicate the median and extent of the interquartile range, with whiskers indicating 1.5 times the IQR or the most extreme value. Units are fluorescence intensity (FI) in the BAMA assay adjusting for the signal measured from a negative control (blank bead)

Study	Estimate	SE	Weight	95% CI
HVTN 122	−0.53	0.38	5.4%	[−1.29; 0.24]
HVTN 98	−0.23	0.30	9.1%	[−0.82; 0.36]
HVTN 96	−0.22	0.24	13.5%	[−0.70; 0.26]
HVTN 204	−0.13	0.15	33.3%	[−0.44; 0.18]
HVTN 505	−0.12	0.17	26.9%	[−0.46; 0.22]
HVTN 205	0.19	0.26	11.7%	[−0.33; 0.71]
**Total**	**−0.13**		**100.0%**	**[−0.31; 0.04]**

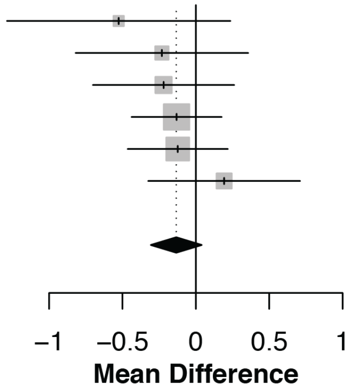

Figure S2. Difference in post-vaccine anti-gp120 IgG comparing trial participants with pre-vaccine anto-gp41 IgG level above and below the median. Estimates and total pooled mean difference were estimated using random effects meta-analysis (see Methods for details). Anti-gp120 IgG was measured using a vaccine matched antigen from protocols where vaccines included gp41, excluding HVTN 106. Forest plot indicates estimated difference for each study, using size of the gray square to indicate study size, and horizontal line to indicate 95% CI.

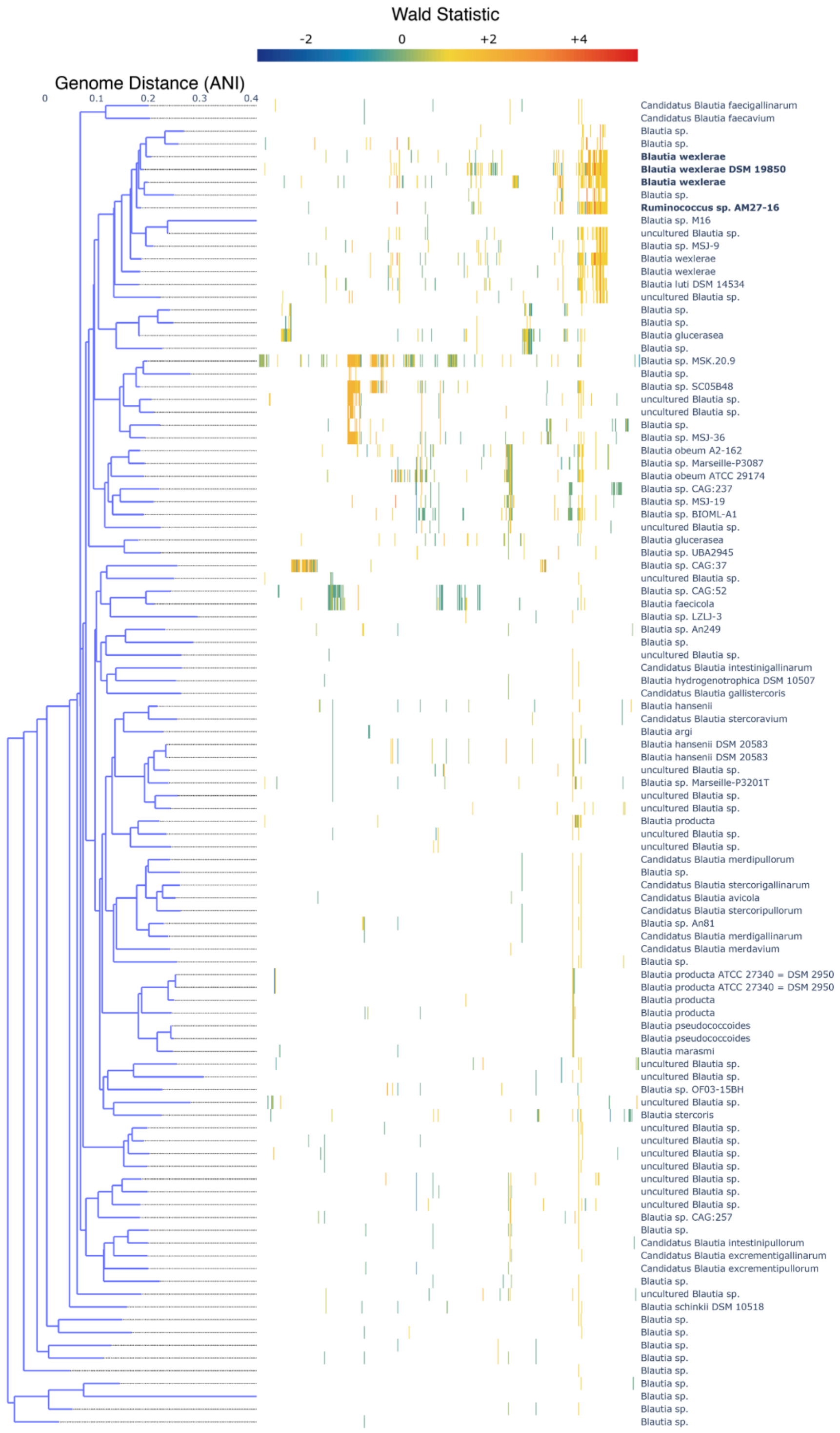

Figure S3. Fine scale mapping of genes identified in CAGs to genomes in the Blautia genus. Genes are mapped to genomes if they share 90% alignment identity at the amino acid level. The genes belonging to CAGs most consistently associated with anti-gp41 IgG pre vaccine have higher positive Wald statistics. Wald statistic indicates the association of each CAG among pre-vaccine gp41 levels in HVTN 106 participants.

Supplemental Tables

Table S1. Participants by Country with Pre-Vaccine anti-gp41 IgG Data Passing QC Requirements

	Trial	**BR**	**HT**	**JA**	**MZ**	**SZ**	**SW**	**TZ**	**TH**	**US**	**ZM**	**ZB**
HVTN	96						80					
HVTN	97					79						
HVTN	98									54		
HVTN	100					200						
HVTN	106						12			65		
HVTN	107				20	50						22
HVTN	111					51		25			21	
HVTN	122									27		
RV	144								219			
HVTN	204	20	9	12		175				123		
HVTN	205									62		
HVTN	505									144		

Country abbreviations:
(BR) Brazil (HT) Haiti (JA) Jamaica (MZ) Mozambique (SZ) South Africa (SW) Switzerland (TZ) Tanzania (TH) Thailand (US) United States (ZM) Zambia (ZB) Zimbabwe

Table S2. Regression Models Comparing Log10-Transformed Pre-Vaccine anti-gp41 IgG in Participants from the United States and South Africa.

	Model 1			Model 2			Model 3		
	**Beta**	**95% CI[1]**	**p-value**	**Beta**	**95% CI[1]**	**p-value**	**Beta**	**95% CI[1]**	**p-value**
age	-0.002	-0.008, 0.004	0.595	-0.002	-0.008, 0.004	0.49	-0.002	-0.008, 0.004	0.511
sex									
Male	-0.107	-0.195, -0.019	0.017	-0.062	-0.153, 0.030	0.188	-0.063	-0.150, 0.025	0.159
country									
United States	-0.214	-0.308, -0.119	<0.001	-0.372	-0.529, -0.214	<0.001	-0.373	-0.527, -0.218	<0.001
trial									
98				0.485	0.208, 0.763	0.001	0.485	0.213, 0.757	<0.001
100				0.237	0.064, 0.411	0.007	0.237	0.067, 0.408	0.006
106				0.529	0.261, 0.797	<0.001	0.528	0.265, 0.792	<0.001
111				0.197	-0.044, 0.438	0.108	0.197	-0.040, 0.433	0.103
122				0.755	0.425, 1.084	<0.001	0.754	0.431, 1.078	<0.001
204				0.407	0.229, 0.585	<0.001	0.407	0.232, 0.581	<0.001
205				0.425	0.156, 0.695	0.002	0.426	0.161, 0.691	0.002
505				0.262	0.018, 0.506	0.036	0.262	0.022, 0.501	0.032
107							0.601	0.369, 0.833	<0.001

[1] CI = Confidence Interval

SI Table 3. Regression Models Comparing Log10-Transformed Pre-Vaccine anti-gp41 IgG Responses in Participants from the United States and South Africa in HVTN 204

	Model 1			Model 2		
	**Beta**	**95% CI[1]**	**p-value**	**Beta**	**95% CI[1]**	**p-value**
sex						
Male	0.097	-0.039, 0.234	0.162	0.099	-0.038, 0.236	0.156
country						
United States	-0.379	-0.517, -0.241	<0.001	- 0.387	-0.535, -0.239	<0.001
age				0.001	-0.008, 0.010	0.763

[1] CI = Confidence Interval

Table S4. Regressions of Vaccine-Matched Mean log10 gp120 MFI by Trial

HVTN 106	estimate	SE	p.value
(Intercept)	-0.75	0.72	0.30
log10 pre-vaccine gp41	0.57	0.22	0.01
male	-0.22	0.23	0.33
age	-0.01	0.01	0.21
**HVTN 122**			
(Intercept)	1.09	1.42	0.45
log10 pre-vaccine gp41	-0.14	0.51	0.78
male	-0.91	0.41	0.04
age	-0.01	0.02	0.56
**HVTN 204**			
(Intercept)	-0.87	0.48	0.07
log10 pre-vaccine gp41	0.02	0.14	0.88
male	0.04	0.16	0.78
age	0.03	0.01	0.01
**HVTN 205**			
(Intercept)	-0.60	0.90	0.51
log10 pre-vaccine gp41	0.20	0.26	0.44
male	0.17	0.27	0.54
age	0.00	0.02	0.99
**HVTN 505**			
(Intercept)	0.13	0.52	0.80
log10 pre-vaccine gp41	-0.11	0.16	0.49
age	0.00	0.01	0.73
**HVTN 96**			
(Intercept)	-0.95	0.75	0.21
log10 pre-vaccine gp41	-0.14	0.23	0.55
male	0.61	0.23	0.01
age	0.03	0.02	0.03
**HVTN 98**			
(Intercept)	2.24	0.83	0.01
log10 pre-vaccine gp41	-0.31	0.27	0.25
male	-0.83	0.27	0.00
age	-0.03	0.01	0.03

HVTN 100	estimate	SE	p.value
(Intercept)	0.01	0.60	0.98
log10 pre-vaccine gp41	0.15	0.15	0.33
male	-0.22	0.16	0.15
age	-0.01	0.02	0.59
**HVTN 107**			
(Intercept)	-1.16	1.01	0.25
log10 pre-vaccine gp41	0.40	0.30	0.19
male	-0.21	0.21	0.31
age	0.01	0.02	0.75
**HVTN 111**			
(Intercept)	0.47	0.82	0.56
log10 pre-vaccine gp41	0.12	0.21	0.57
male	-0.52	0.21	0.02
age	-0.02	0.02	0.35
**HVTN 97**			
(Intercept)	1.14	0.98	0.25
log10 pre-vaccine gp41	-0.24	0.26	0.36
male	-0.05	0.27	0.86
age	-0.03	0.03	0.45
**HVTN 144**			
(Intercept)	-0.85	0.32	0.01
log10 pre-vaccine gp41	0.35	0.13	0.01
male	0.11	0.14	0.45

Table S5. Beta-Binomial Regression Results of Taxa Associated with pre-Vaccine anti-gp4 IgG

Taxa	Taxa ID	Estimate	95% CI	p-value	q-value
Ruminococcus sp. AM22-13 (high average nucleotide identity to Blautia wexlerie)	2292074	2.5	[1.4 , 3.5]	6E-05	3E-04
Ruminococcus sp. AM27-16 (high average nucleotide identity to Blautia wexlerie)	2293192	0.9	[0.5 , 1.3]	2E-04	8E-04
Blautia wexlerae	418240	0.9	[0.5 , 1.4]	5E-04	2E-03
Coprococcus sp. OM04-5BH	2293093	1.2	[0.6 , 1.8]	7E-04	3E-03
Acetobacterium sp. KB-1	2184575	3.3	[1.6 , 5]	7E-04	3E-03
Bilophila sp. 4_1_30	693988	1.5	[0.7 , 2.2]	9E-04	4E-03
Bilophila	35832	1.1	[0.5 , 1.6]	1E-03	5E-03
Bilophila wadsworthia	35833	1.1	[0.5 , 1.8]	1E-03	5E-03
Anaeroglobus geminatus	156456	2.6	[1.2 , 4.1]	1E-03	7E-03
Akkermansia sp. KLE1797	1574264	-13.5	[-21.7 , -5.4]	3E-03	1E-02
Coprobacillus sp. AM32-11LB	2293080	2.0	[0.8 , 3.2]	3E-03	1E-02
Bacillales	1385	0.3	[0.1 , 0.5]	3E-03	1E-02
Lachnospira pectinoschiza	28052	1.2	[0.5 , 2]	3E-03	1E-02
Pseudoflavonifractor sp. An44	1965635	1.3	[0.5 , 2.1]	3E-03	1E-02
Clostridium beijerinckii	1520	3.5	[1.3 , 5.7]	4E-03	2E-02
Anaerobiospirillum thomasii	179995	1.7	[0.6 , 2.7]	4E-03	2E-02
Bacteroides sp. AM18-9	2292940	-7.1	[-11.7 , -2.5]	5E-03	2E-02
unclassified Erysipelotrichaceae	544447	1.5	[0.5 , 2.5]	5E-03	2E-02
Lactonifactor	420345	3.2	[1.1 , 5.3]	5E-03	2E-02
Synergistes jonesii	2754	0.9	[0.3 , 1.6]	6E-03	3E-02
Aquitalea sp. MWU14-2217	2480983	1.7	[0.6 , 2.8]	6E-03	3E-02

Rikenellaceae bacterium	2049048	1.0	[0.3 , 1.7]	6E-03	3E-02
Blautia sp. Marseille-P3201T	1907659	0.9	[0.3 , 1.5]	7E-03	3E-02
Brachyspira hyodysenteriae	159	1.5	[0.5 , 2.5]	8E-03	4E-02
Ruminococcus sp. TM09-4	2293244	0.8	[0.2 , 1.3]	8E-03	4E-02
Faecalibacterium sp. AM43-5AT	2302957	0.8	[0.2 , 1.3]	9E-03	4E-02
Eubacterium sp. 3_1_31	457402	1.7	[0.5 , 3]	1E-02	4E-02
Peptoniphilus sp. BV3C26	1111134	-4.5	[-7.7 , -1.3]	1E-02	5E-02
Coprobacillus sp. AF35-8	2293070	1.3	[0.4 , 2.3]	1E-02	5E-02
Pseudoflavonifractor sp. An187	1965578	3.3	[0.9 , 5.7]	1E-02	5E-02
[Clostridium] spiroforme	29348	0.6	[0.2 , 1.1]	1E-02	5E-02
